# A guide to UFMylation, an emerging posttranslational modification

**DOI:** 10.1111/febs.16730

**Published:** 2023-02-08

**Authors:** David Millrine, Joshua J. Peter, Yogesh Kulathu

**Affiliations:** ^1^ Medical Research Council Protein Phosphorylation & Ubiquitylation Unit (MRC‐PPU), School of Life Sciences University of Dundee UK

**Keywords:** endoplasmic reticulum, ligase, protease, proteostasis, ubiquitin‐like modifier, UFM1

## Abstract

Ubiquitin Fold Modifier‐1 (UFM1) is a ubiquitin‐like modifier (UBL) that is posttranslationally attached to lysine residues on substrates via a dedicated system of enzymes conserved in most eukaryotes. Despite the structural similarity between UFM1 and ubiquitin, the UFMylation machinery employs unique mechanisms that ensure fidelity. While physiological triggers and consequences of UFMylation are not entirely clear, its biological importance is epitomized by mutations in the UFMylation pathway in human pathophysiology including musculoskeletal and neurodevelopmental diseases. Some of these diseases can be explained by the increased endoplasmic reticulum (ER) stress and disrupted translational homeostasis observed upon loss of UFMylation. The roles of UFM1 in these processes likely stem from its function at the ER where ribosomes are UFMylated in response to translational stalling. In addition, UFMylation has been implicated in other cellular processes including DNA damage response and telomere maintenance. Hence, the study of UFM1 pathway mechanics and its biological function will reveal insights into fundamental cell biology and is likely to afford new therapeutic opportunities for the benefit of human health. To this end, we herein provide a comprehensive guide to the current state of knowledge of UFM1 biogenesis, conjugation, and function with an emphasis on the underlying mechanisms.

## Graphical abstract

UFM1 is a ubiquitin‐like modifier (UBL) that is important to maintain homeostasis at the endoplasmic reticulum and mutations in this pathway lead to neurodevelopmental disorders and multiple pathologies. Being the most recently discovered UBL, we are only now beginning to understand the mechanisms and functions of this enigmatic modifier. In this review, we provide a comprehensive guide to the mechanisms and biological function of protein UFMylation.

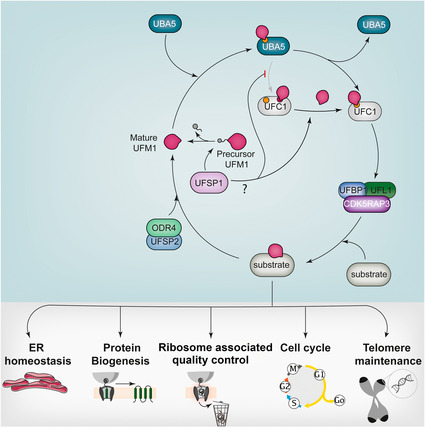

**Abbreviations**
Aim: ATG8‐interacting motif
ASC1: Asc‐type amino acid transporter‐1
ATG8: autophagy‐related protein‐8
CDK5RAP3: CDK5 regulatory subunit associated protein 3
Cryo‐EM: cryogenic electron microscopy
ER: endoplasmic reticulum
ERAD: endoplasmic reticulum‐associated protein degradation
ISG15: interferon stimulated gene‐15
NEDD8: neural precursor cell expressed developmentally down‐regulated protein‐8
ODR4: odorant response abnormal protein‐1
PCI domain: proteosome component domain
PTM: posttranslational modification
RPL26: 60S ribosomal protein L26
SEMD: Sohat‐type spondyloepimetaphyseal dysplasia
TRIP4: thyroid hormone receptor interactor‐4
UB: ubiquitin
UBA5: UBL activating enzyme‐5
UBL: ubquitin‐like protein
UFBP1: UFM1 binding protein‐1
UFC1: UFM conjugating enzyme‐1
UFL1: UFM1 specific ligase‐1
UFM1: ubiquitin fold modifier‐1
UFSP1: UFM1 specific protease‐1
UFSP2: UFM1 specific protease‐2
UPR: unfolded protein response
WH domain: Wing‐Helix domain

## Introduction

Ubiquitin and ubiquitin‐like modifiers (UBLs) are post‐translationally attached to proteins and play essential roles in regulating eukaryotic biology [1]. UFM1 is a ubiquitin‐like modifier that was discovered only in 2004 [2]. Despite limited homology at the sequence level (< 21%), ubiquitin and UFM1 share a similar tertiary structure and a conceptually similar enzymatic pathway (i.e. involving E1, E2, and E3 enzymes) that culminates in the covalent attachment of UFM1 to substrate lysine residues via its C‐terminal glycine [2]. Like ubiquitin, UFM1 can also be assembled into polymer chains as it contains five lysine residues. However, these polyUFM1 chains appear to be predominantly linked via K69 [3, 4]. In contrast to protein ubiquitylation, which is regulated by hundreds of enzymes with overlapping function, UFMylation is noted for its remarkably limited and non‐redundant cellular machinery that exhibits a high level of specificity. Only a handful of enzymes, all ubiquitously expressed, are reported to mediate UFM1 maturation, activation, attachment, and removal. These are UBA5, UFC1, and UFL1 identified as the E1, E2, and E3 enzymes respectively, while UFSP1 and UFSP2 are the UFM1‐specific proteases [5, 6, 7, 8, 9, 10, 11]. Other proteins linked to UFMylation play various scaffolding or targeting functions (UFBP1, CDK5RAP3, ODR4) that ensure enzyme activity is appropriately directed or segregated into relevant cellular compartments [4, 12, 13, 14, 15]. Of note, UFM1 and the components of this pathway are evolutionarily conserved and present in nearly all eukaryotes [12].

While mutations in components of the UFM1 pathway are associated with several diseases, the mechanisms and functional importance of the UFM1 pathway have remained elusive. The UFM1 pathway has attracted significant recent interest following a series of recent landmark studies that have delivered important biological insights into this poorly understood UBL. Key among them is the observation that the 60S ribosomal subunit RPL26 (uL24) is one of the main targets of UFMylation in cells [16, 17]. Further, translational stalling resulting in Sec61 translocon clogging stimulates UFMylation of endoplasmic reticulum (ER)‐resident but not cytosolic ribosomes [13, 18]. These observations have led to the conjecture that UFMylation operates at the ER in concert with the ribosome quality control (RQC) pathway to assist cells in dealing with potentially hazardous non‐functional ribosomes. Yet despite substantial recent progress, there are significant knowledge gaps in our understanding of protein UFMylation that provide rich opportunities for basic and translational research in the coming decade.

In this review, we provide a comprehensive overview of the molecular players and our present understanding of the mechanisms by which they catalyse UFMylation. We focus on the key studies that shed light on the mechanisms of UFMylation, which have dramatically shaped our understanding of this fascinating PTM. As UFMylation is a dynamic reversible process in cells, we discuss how proteases regulate this PTM. We then discuss the role of UFMylation in ER homeostasis and the impact of mutations and loss of UFMylation in experimental models and in human disease.

## Molecular players catalysing UFMylation

**Fig. 1:**
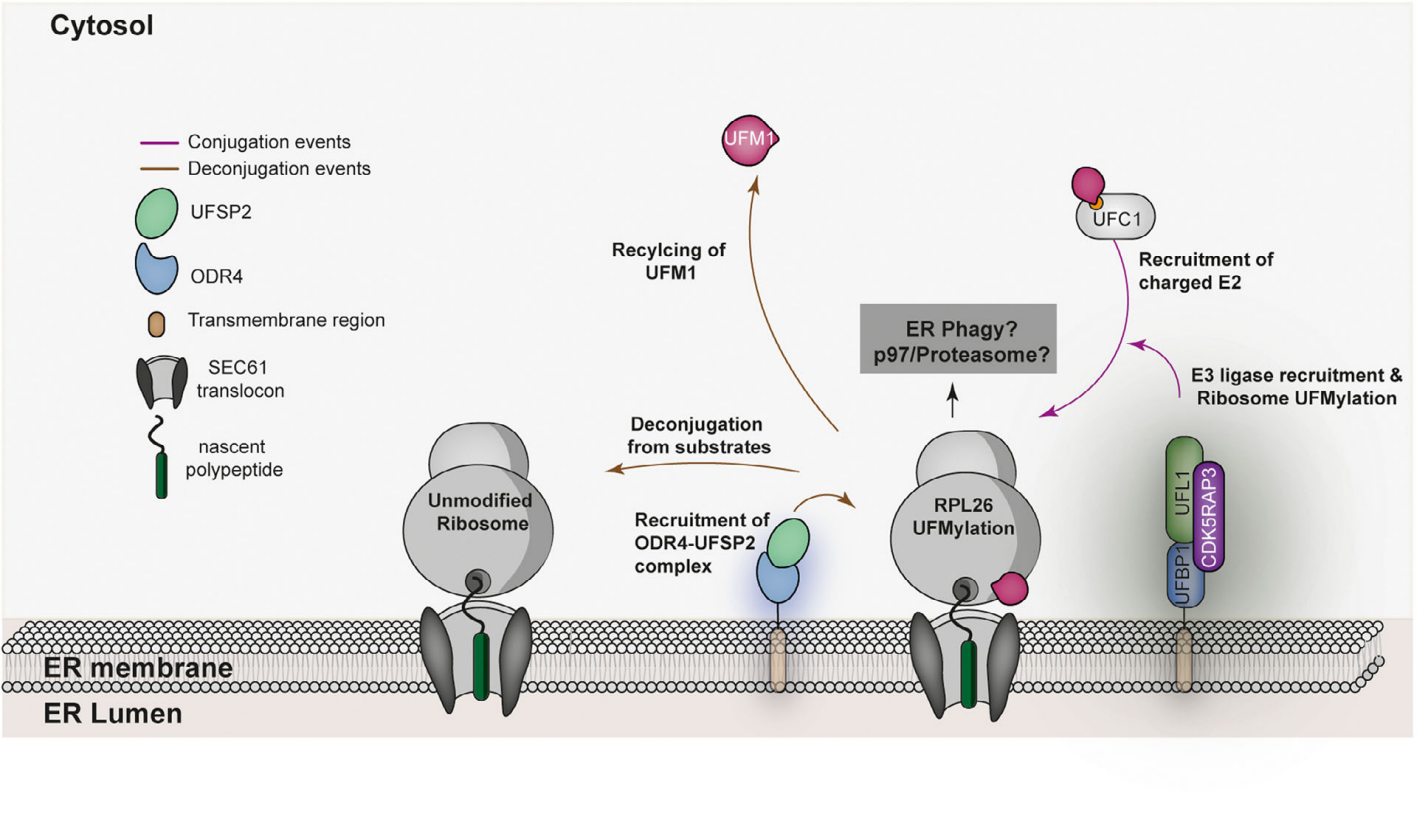
Schematic overview of the UFM1 pathway. Translational stalling triggers the posttranslational modification of the ribosomal subunit RPL26 with UFM1 via a classical thiolation, trans‐thiolation, and ligation pathway involving the enzymes UBA5, UFC1, and UFL1‐UFBP1. UFL1 functions as a scaffold‐type E3 ligase and is stabilized and anchored to the ER via an interaction with UFBP1. The pathway is negatively regulated by proteolysis through the action of the ER‐membrane‐associated ODR4‐UFSP2 complex. UFMylation of ribosomes is linked to a specialized autophagy of the ER‐membrane, ER‐phagy [49].

Substrate UFMylation requires a sequential pathway analogous to that of protein ubiquitylation involving a cascade of E1 activating, E2 conjugating, and E3 ligating enzymes [2, 19]. In contrast to the ubiquitin system however, there is only a single E1 (UBA5), E2 (UFC1) and E3 (UFL1) that work to catalyse an isopeptide bond between the C‐terminal glycine of UFM1 and the ε‐amino group of the substrate lysine. Briefly, the C‐terminal glycine is adenylated by UBA5 in an ATP‐dependent reaction that releases pyrophosphate (PPi) [2]. The C‐terminal glycine of adenylated UFM1 is next subject to nucleophilic attack by the UBA5 catalytic cysteine (C250) (Note: all numbering refers to human enzymes unless otherwise stated) resulting in the formation of a thioester bond (thiolation/thioesterification) [20, 21, 22]. UFM1 is next transferred to the catalytic cysteine (C116) of UFC1 in a process termed trans‐thiolation [23]. From here, UFM1 is covalently conjugated to substrate lysine residues through a process requiring the E3 ligase component UFL1 [4, 24]. Together, these processes form a classical thiolation, trans‐thiolation, and ligation pathway that is observed throughout UBL systems (Fig. 1) [25]. Of note, important structural characteristics ensure fidelity of the conjugation process and exclude undesirable crosstalk with other UBL pathways. We first discuss this core machinery and the molecular characteristics that are in several regards unique among UBL conjugating enzymes.

### Cleave to activate – role of proteases in activating UFMylation

The ubiquitin system is regulated by 99 deubiquitylating enzymes (DUBs) that are integral to diverse biological processes including cell survival, immune responses, DNA damage repair, intracellular transport, endosomal sorting, autophagy, and the cell cycle, among many other possible examples [26, 27]. The analogous enzymes regulating protein UFMylation are the UFM1‐specific proteases UFSP1 and UFSP2 [28]. Like most UBLs, *UFM1* is expressed in a precursor form that requires proteolytic maturation to be active. Therefore, an important function of UFSPs is the generation of a free pool of mature UFM1 through proteolysis of the UFM1 precursor. This involves the removal of a serine^84^‐cysteine^85^ di‐peptide to expose the C‐terminal glycine of UFM1 that is required for conjugation to substrates [28]. Until recently, UFSP1 was thought to be catalytically inactive in humans due to its mistaken annotation as an ‘Inactive UFM1 specific protease’. Two concurrent studies have now identified active UFSP1 translated from a non‐canonical CTG start codon in human cells [13, 29]. Consequently, human cell lines lacking both UFSPs (*UFSP1* ^*−/−*^ */UFSP2* ^*−/*^) show a complete loss of UFMylation [13]. Importantly, substrate UFMylation is rescued through over‐expression of mature (UFM1^1–83^) but not precursor (UFM1^1–85^) UFM1 [13]. Hence, UFM1^Ser84‐Cys85^ cleavage is critical for a functional UFM1 pathway [13, 28].

In addition to precursor maturation, UFSP1 and UFSP2 regulate UFMylation of substrates. For instance, UFMylation of the ribosomal subunit RPL26, which is dramatically upregulated upon loss of UFSP2, is unaffected by UFSP1 deficiency. Instead, a marked increase in UFM1 modification of UFC1 is observed in cells lacking UFSP1 [13]. Interestingly, this modification occurs at K122 (located at the +6 position relative to the catalytic C116). Studies on the ubiquitin E2 enzyme UBE2S have suggested that E2 auto‐modification on a similarly positioned lysine residue is auto‐inhibitory and reduces the efficiency of ubiquitin transfer from the E1 to the E2 catalytic site via steric hindrance [30]. This mechanism appears highly conserved as up to 25% of E2 enzymes have a lysine residue at this position. Whether this is true of UFC1 is unclear, however, it is speculated that UFSP1 may relieve such negative regulation to regulate the kinetics of UFM1 transfer [13]. Hence UFSP1 may act at two levels to activate UFMylation, first in the proteolytic maturation of pro‐UFM1 and secondly in the cleavage of a putative auto‐inhibitory modification of UFC1.

### Determinants of UFSP substrate specificity

The UFSPs are noted for their homology to ZUFSP, a recently discovered DUB that has marked sequence divergence from established DUB families. However, there is currently no evidence that ZUFSP has any activity towards the UFM1 precursor or conjugates [31, 32]. Activity‐based probes made through the attachment of reactive warheads such as propargylamine, chloroethylamine, and bromoethylamine to the C‐termini of UBLs have been invaluable tools to trap covalent complexes of the UBL together with its cognate proteases [33, 34]. Both UFSPs react with activity‐based probes (e.g. UFM1‐vinylmethylester (VME)) and are active against UFM1 model substrates but not those of other UBLs (ISG15, ATG8, NEDD8, UB) [28, 33, 35]. While UFM1 probes using commonly used warheads showed either poor reactivity or cross‐reactivity with other UFMylation enzymes, the recent development of a α‐chloroacetyl linked probe showed selective targeting of UFSP2 [36]. In addition to the reactive warhead used, specificity is also driven by UFM1. Indeed, specificity of UFMylation enzymes for UFM1 over other UBLs is impressive and involves several factors including unique complementary electrostatic surfaces between the enzyme‐UBL interfaces or specific amino acid differences within the UBLs themselves [37, 38]. For example, UFM1 is the only UBL to possess a valine residue proximal to the C‐terminal glycine and this difference prevents deubiquitinating enzymes from recognizing UFM1 [31, 37]. These small but functionally significant adaptations prevent unwanted crosstalk between different UBL pathways and ensure specificity and fidelity within the UFM1 system [38].

Despite close structural homology between the catalytic domains, UFSP1 and UFSP2 have distinct substrate preferences. We discuss two factors that may influence UFSP substrate preference. First, are structural features inherent to the catalytic site. Both UFSPs share a thiol protease catalytic mechanism with a conserved catalytic triad composed of cysteine, aspartate, and histidine residues together with a proximal tyrosine residue that participates in oxyanion hole formation [37, 39, 40]. While at first glance the catalytic regions of UFSP1 and UFSP2 have a closely related tertiary structure, they are not identical. Notable differences between the UFSPs are observed in three flexible loops of regulatory significance (Regulatory (R), Upstream (U), and Neighbouring (N) loops) [39]. The most important of these, the R loop, directly interfaces with the UFM1 C‐terminal residues while the two additional loops sit adjacent to the catalytic triad. Evolutionary divergence has resulted in a series of amino acid substitutions, insertions, and deletions, that have altered the length and/or composition of these and other regulatory domains in UFSP1 when compared with UFSP2. For example, UFSP2 has an extended α‐helix harbouring the catalytic cysteine [39]. Evidence for the importance of these subtle changes comes from studies of chimeric proteins whereby exchange of these regulatory loops influences the activity of UFSPs on model substrates *in vitro*. For instance, UFSP2 harbouring the R loop of UFSP1 is rendered catalytically inactive but may be rescued through receipt of the UFSP1 N‐loop [39]. Hence, several loops within the UFSP catalytic domain collectively support proteolysis [37, 39, 40]. Further, the presence of these loops modulating proteolytic activity suggests that the UFSPs are regulated by allosteric mechanisms.

**Fig. 2:**
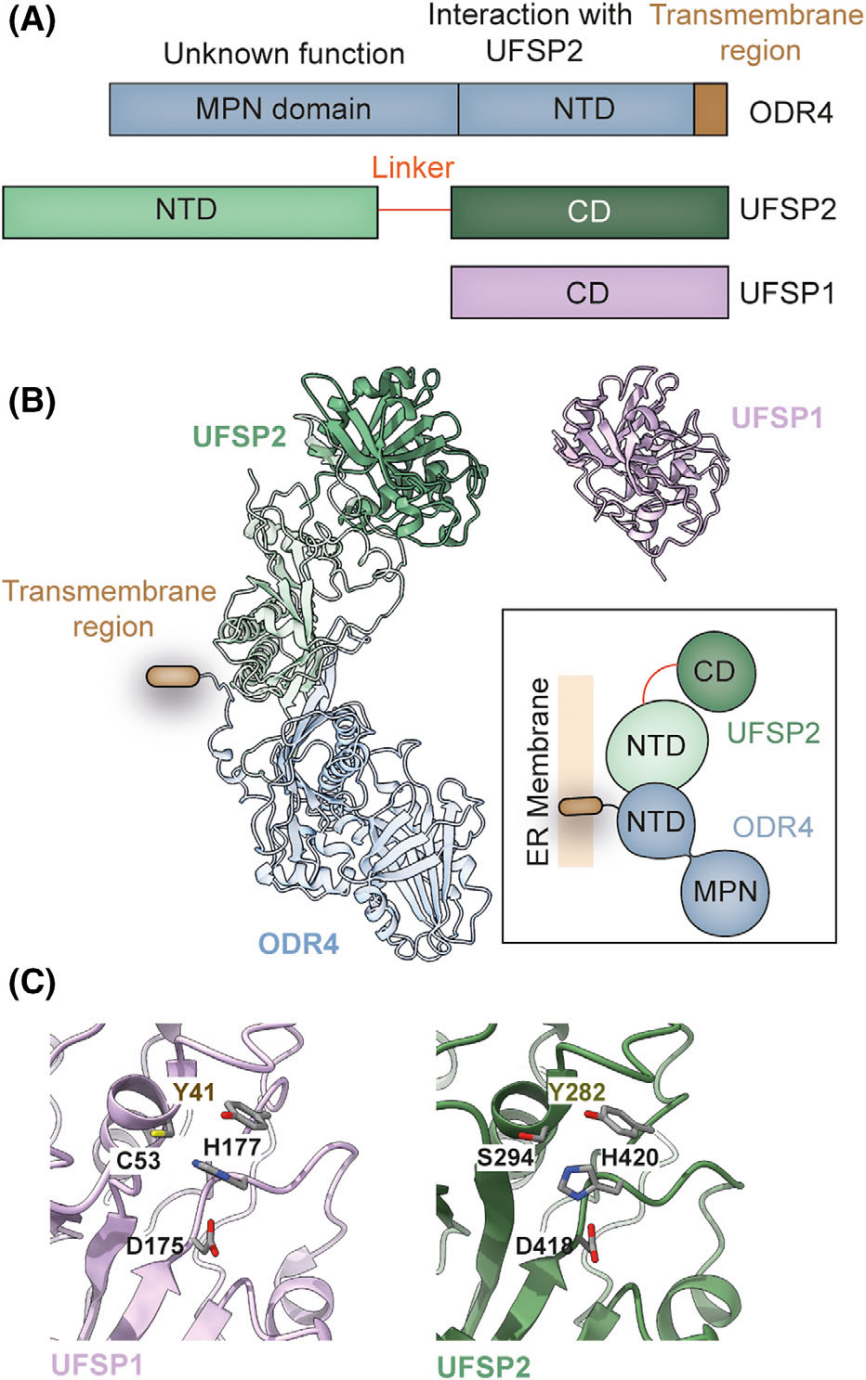
Features of UFSP proteases. (A) Schematic comparing key domains of human UFSP1, ODR4 and UFSP2. UFSP1 lacks the N‐terminal region responsible for UFSP2‐ODR4 interaction (NTD, N‐terminal domain; CD, catalytic domain; MPN, Mpr1, Pad1 N‐terminal). (B) Predicted structure of the UFSP2‐ODR4 complex (left) and crystal structure of murine UFSP1 (right) (PDB ID: 2Z84) shown in cartoon representation. Inset contains a schematic showing the mode of interaction between UFSP2 and ODR4. (C) Enlarged view of the catalytic cleft of murine UFSP1 (left) (PDB ID: 2Z84) and human UFSP2 (right) (PDB ID: 3OQC) shown in cartoon representation. Key catalytic residues are highlighted and shown as sticks. Catalytic cysteine of UFSP2 is mutated to serine in the crystal structure. Structures depicted in (B) and (C) were analysed and visualized using ucsf chimerax [91, 92].

A second factor influencing UFSP substrate preference is subcellular compartmentalization. Components of the UFMylation pathway are either localized in the cytosol or associated with the ER [13]. The UFSPs show distinct subcellular localization with UFSP1 being predominantly cytosolic whereas UFSP2 localizes to the ER by virtue of its association with the ER‐transmembrane protein, ODR4 [13, 14]. This association with ODR4 is essential for UFSP2 localization to the ER as it does not itself possess a transmembrane domain. The interaction also serves a mutually stabilizing function as ODR4 protein levels are markedly reduced in cells lacking UFSP2, and UFSP2 protein levels are likewise diminished by the absence of ODR4 [13]. Interestingly, structure predictions reveal that ODR4 adopts a metalloprotease (MPN) fold but is a pseudoenzyme as it lacks the catalytic residues. Alphafold predictions reveal human UFSP2 to have a bilobed architecture with a C‐terminal catalytic domain that is similar to UFSP1 and an additional N‐terminal domain [41]. Importantly, modelling using Alphafold suggests that UFSP2 and ODR4 may directly interact to form a complex via three parallel β‐sheets in the UFSP2 N‐terminal domain [13] (Fig. 2). Intriguingly, *Caenorhabditis elegans* UFSP2 is expressed as a single polypeptide sequence which also contains this MPN domain present in ODR4 [42]. These *in silico* predictions of human UFSP2‐ODR4 interaction are also consistent with experimental evidence demonstrating that UFSP2 mutants lacking this N‐terminal region fail to associate with ER‐bound UFBP1 [39]. This differential sub‐cellular localization also establishes a division of labour wherein UFSP1 acts on the predominantly cytosolic pro‐UFM1 and auto‐UFMylated UFC1, whereas ER‐localized UFSP2 is better positioned to act on UFMylated ribosomes [13].

While UFSP1 and UFSP2 exhibit distinct substrate preferences, at present no quantitative comparisons of UFSP activity have been made making it difficult to assess the relative contributions of differences in structure and cellular localization to substrate preference. Qualitative gel‐based assays have generally advanced the idea that UFSP1 as the more active peptidase with activity against the UFM1 precursor [13, 28]. However, it is important to note that these studies have only evaluated UFSP2 in isolation and not in complex with ODR4, which is significant since ODR4 may allosterically regulate UFSP2 catalytic activity. Future studies addressing substrate recognition and the significance of UFC1 auto‐modification at K122 will reveal mechanistic insights into how UFMylation is regulated.

### UBA5 – a variation on a theme

**Fig. 3:**
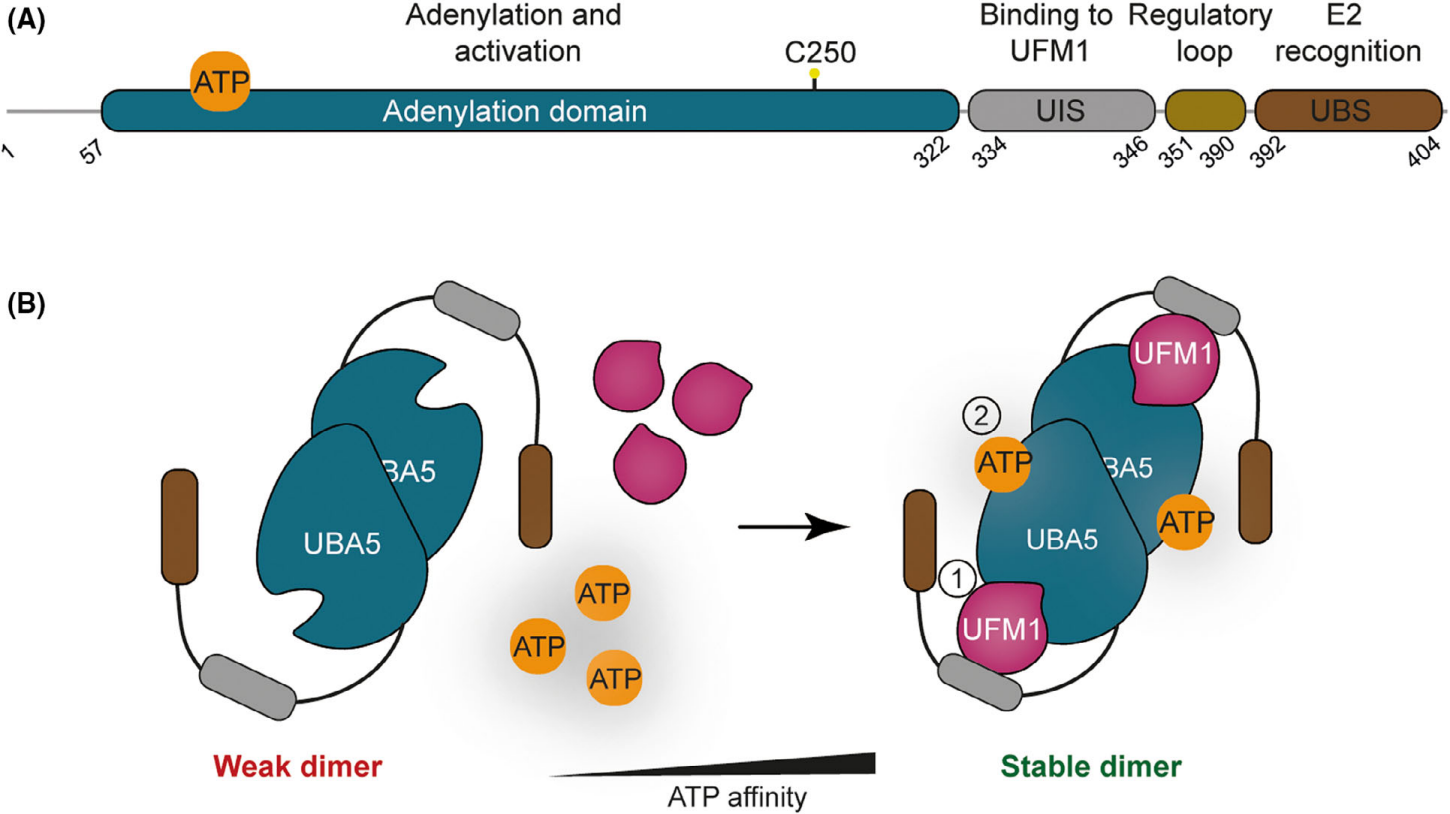
Mechanism of UFM1 activation. (A) Schematic showing key domains of UBA5. (B) Activation of UFM1 in *trans*. UFM1 binds one UBA5 monomer and is activated by the other. UBA5 is a weak dimer in solution. UFM1 binding stabilizes the UBA5 dimer which is accompanied by an increase in its affinity for ATP. UFM1‐interacting sequence (UIS) and the UFC1‐Binding Sequence (UBS) are shown in grey and brown respectively.

Following UFM1 maturation, UBA5 sits at the apex of the UFMylation pathway. Insights into the mechanism of UBA5 come from several structural studies of different UBA5 complexes that represent distinct steps in the catalytic cycle. The structure of UFM1‐bound UBA5 in its pre‐activation state reveals a trans‐binding mechanism that necessitates UBA5 dimerization. By itself, UBA5 only weakly dimerizes. However, when complexed with UFM1, the dimer conformation stabilizes and increases affinity for ATP [21]. Hence, the UFM1 activating complex is comprised of two molecules of UFM1 and two molecules of UBA5 that form an interlocking structure wherein each UFM1 molecule contacts both UBA5 monomers using different interaction surfaces [20]. These interfaces are the UBA5 Adenylation domain (UBA5^57–329^), which contains the catalytic cysteine contributing to UFM1 activation, and the UFM1‐interacting sequence (UIS) (UBA5^338–346^) that mediates non‐covalent UFM1‐UBA5 interactions (UIS). Mutations that disrupt a salt bridge linking the two UBA5 monomers render UBA5 unable to dimerize. However, introducing charge complementing mutations where each mutant in isolation is unable to dimerize, but can form heterodimers when combined has been key to reveal the reciprocal requirement for adenylation and UIS domains from two UBA5 monomers for UFM1 activation [20]. UFM1 therefore binds one UBA5 monomer and is activated by the other [20]. This unusual trans‐binding mechanism is unique among UBL E1 enzymes (Fig. 3).

### E1 to E2 transfer – transthiolation in trans

Following activation, UFM1 is transferred from UBA5 to the catalytic Cys of UFC1, UFC1^C116^ via a trans‐thiolation reaction involving interactions between a UBA5‐UFM1 dimer and UFC1. Intriguingly, over‐expression of UBA5 reverses the flow favouring the transfer of activated UFM1 from UFC1 to UBA5, hinting at reversibility of E2 transfer. The equilibrium of this exchange appears to be dictated by cellular levels of free UFM1 with an excess favouring UBA5‐to‐UFC1 trans‐thiolation [43]. In the canonical direction (E1 to E2), UFC1 engages a tetrameric complex comprised of two molecules of UFM1 and two molecules of UBA5, using a similar trans‐binding mechanism requiring interaction with one UBA5 monomer and receipt of activated UFM1 from the other [20]. A short amino acid sequence, the UBS (UFC1‐Binding Sequence; UBA5^381–404^), has been defined as the minimal requirement for UBA5‐UFC1 interactions while a slightly larger region is required for effective trans‐thiolation (UBA5^264–404^) [44]. This contrasts with the majority of E1 enzymes that have a much larger domain for interacting with the E2 [1, 45]. Importantly, the UBA5^UBS^ interaction occurs at the opposite face to the catalytic site of UFC1 and forms a unique interface that is not compatible with other E2 enzymes when modelled *in silico* [23]. Additional amino acid residues required for trans‐thiolation include a short linker region (UBA5^347–377^) that is dispensable for the intrinsic functioning of UBA5. In a non‐typical E1‐E2 relationship, UBA5 ‘donates’ this linker sequence to UFC1 to facilitate desolvation of the catalytic site (UFC1^C116^), which is unusually acidic due to the absence of a canonical regulatory loop observed in other E2 enzymes. In the absence of this UBA5 donor sequence, UBA5 is only able to transfer UFM1 onto UFC1 under alkaline conditions (pH > 7.5) [23]. This unusual reciprocal relationship is mirrored at the physiological level in reports of early developmental encephalopathy linked to mutations in the UBA5 linker region (UBA5^A371T^) (Table 1) [46]. Alanine^371^ contacts the UFC1 active site and is essential for the desolvation process that exposes the UFC1 catalytic cysteine to nucleophilic attack [23].

**Table 1:** Reported pathogenic mutations in UFM1 pathway components. Mutations are annotated as reported but may differ between studies according to reference sequence used by the author. Compound mutation refers to instances where more than one mutation is linked to morbidity. Phenotypes are intended as a guide to the reported phenotype. ‘Neurodevelopmental’ typically includes, but is not limited to, symptoms of epilepsy. Refer to listed references for detailed summary.

Mutation	Notes	Gene	Phenotype	Reference
H428R	Catalytic histidine	*UFSP2*	Di Rocco‐type Buekes dysplasia	[79]
D418A	Catalytic asparagine	*UFSP2*	Di Rocco‐type Buekes dysplasia	[58]
Y290H	Oxyanion hole tyrosine	*UFSP2*	Buekes dysplasia	[59]
V115E	UFSP2 N‐terminal region	*UFSP2*	Neurodevelopmental	[80]
A81C	C‐terminal	*UFM1*	Neurodevelopmental	[47]
R23G	N‐terminal	*UFM1*	Neurodevelopmental	[47]
3 bp deletion	Non‐coding/promoter region	*UFM1*	Neurodevelopmental	[84, 85, 86]
Intronic (408+1G>A)		*UFBP1*	Musculoskeletal	[82]
T106I	TAK motif/catalytic site	*UFC1*	Neurodevelopmental	[47]
R11W		*UBA5*	Neurodevelopmental	[87]
Y53F		*UBA5*	Aicardi syndrome (epilepsy)	[88]
A371V	Compound mutation	*UBA5*	Epilepsy	[72, 89]
A371T	Compound mutation (R55H)	*UBA5*	Neurodevelopmental	[46]
Intronic (684G>A)	mRNA splicing	*UBA5*	Epilepsy	[89]
R246X	Premature stop codon	*UBA5*	Neurodevelopmental	[90]
K310E		*UBA5*	Neurodevelopmental	[90]
V260M	Compound mutation	*UBA5*	Neurodevelopmental	[72]
M57V	Compound mutation	*UBA5*	Neurodevelopmental	[72]

Another distinguishing feature of UFC1, also seen in a few other UBL E2s, is an N‐terminal helix extending from the core UBC domain. Intriguingly, UFC1 lacking this region shows higher intrinsic lysine reactivity and increased UFMylation of RPL26, suggesting inhibitory roles for this helical extension [4]. Mechanistically, the N‐terminal helix may serve a stabilizing function [23]. Overall, at least four distinguishing characteristics of the E1 and E2 ensure UFM1 pathway fidelity: (a) An unusual mode of UFM1 activation by UBA5 and trans‐thiolation that necessitates UBA5 dimerization; (b) unique complementarity between a hydrophobic pocket in the UBA5^UBS^ and UFC1 surfaces opposite to the catalytic cysteine [23, 44]; (c) regions of UBA5 that facilitate desolvation of the UFC1 catalytic site [23, 44]; and (d) the N‐terminal helix of UFC1 that may serve a stabilizing function [4, 23].

### Transfer of UFM1 from UFC1 to substrate by an atypical ligase complex

**Fig. 4:**
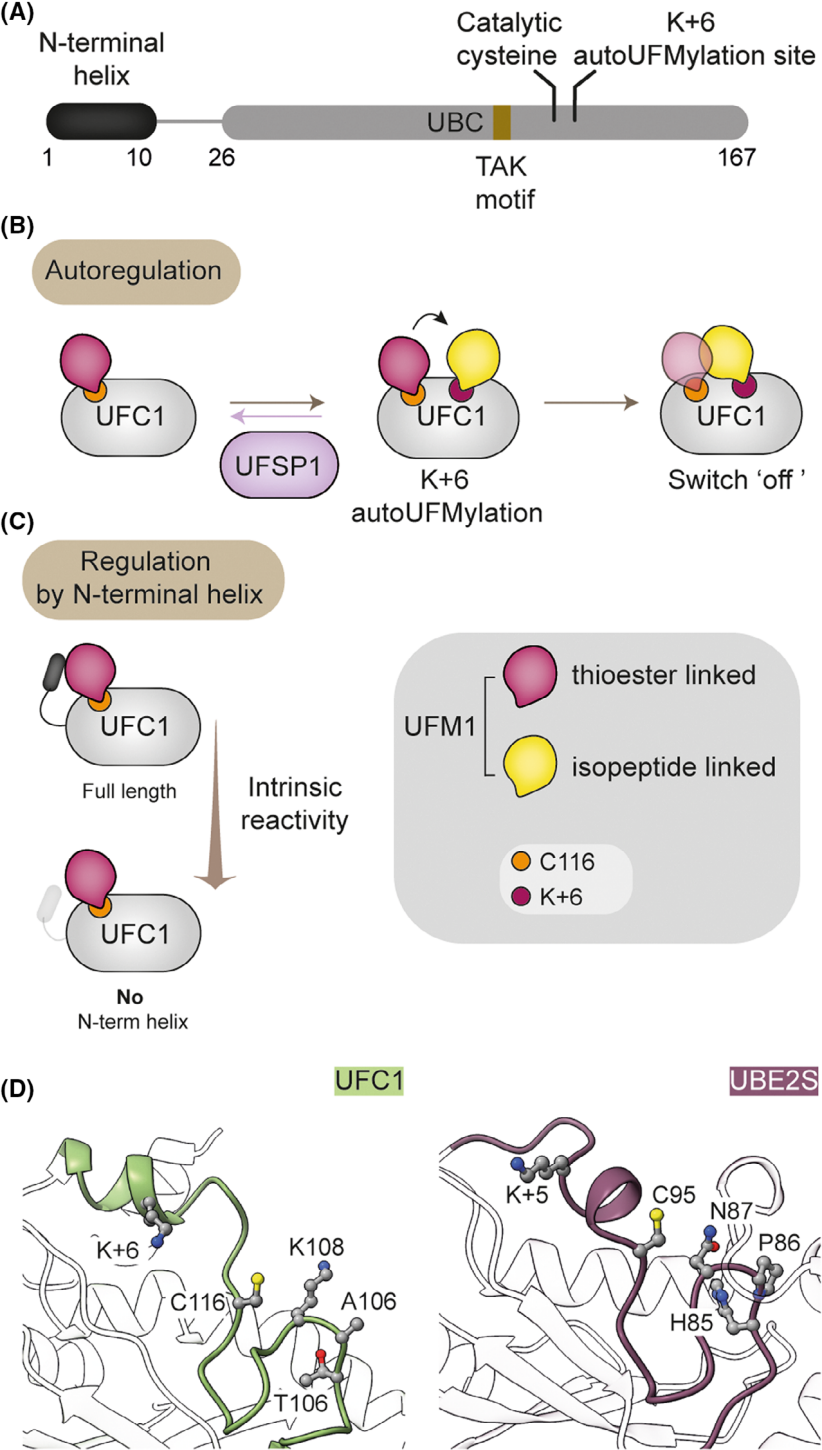
Distinguishing characteristics of UFC1. (A) Schematic showing the key domain features of UFC1. The K+6 auto‐UFMylation site situated six residues downstream of the catalytic site is highlighted. (B) Speculative model for the regulatory function of the K+6 auto‐modification. UFSP1 catalyses removal of UFM1 from K+6 lysine residue. (C) The N‐terminal helix of UFC1 negatively regulates intrinsic reactivity as UFC1 lacking this helix shows increased reactivity. (D) Catalytic residues and K+6 of UFC1 (PDB ID: 2Z6O) compared with UBE2S (PDB ID: 1ZDN). The structures depicted in (D) were analysed and visualized using ucsf chimerax [91, 92].

The final step is the transfer of UFM1 from UFC1 and covalent ligation onto substrate lysine residues, which requires the E3 enzyme, UFL1. As discussed in the preceding sections, pioneering work mainly from the Wiener lab has revealed the mechanism of the E1 and E1‐E2 steps of the pathway. However, UFC1 has other distinctive features when compared to other E2s and how it enables the transfer of UFM1 onto substrates is not fully understood (Fig. 4). For instance, canonical E2 enzymes contain a highly conserved histidine‐proline‐asparagine (HPN) motif where the asparagine residue functions to stabilize the thioester bond prior to catalysis. In UFC1, this HPN motif is replaced by a threonine‐alanine‐lysine (TAK) motif whose function is less clear. Importantly, mutations within the TAK motif impair E2 function and are associated with human disease [4]. A threonine to isoleucine substitution (T106I) in the TAK motif is observed in individuals with neurodevelopmental and musculoskeletal abnormalities [47]. Studies using *in vitro* reconstitution systems demonstrate that the disease‐causing mutant reduces but does not fully abolish the activity of UFC1, suggesting a possible explanation for the survivability of these individuals [4].

How UFL1 functions is poorly understood as its structural domains lack homology to well‐characterized ubiquitin E3 ligases. Furthermore, its structure has been difficult to ascertain due to problems with stable expression and purification of recombinant UFL1 [4]. Since genetic knockout of UFL1 results in a complete loss of UFMylation in cells, it is widely accepted as the sole E3 enzyme. However, direct biochemical evidence demonstrating robust E3 activity by UFL1 was lacking until recently. A breakthrough was the recognition that UFL1 requires an interacting partner, UFBP1, sometimes referred to as DDRGK1 [4]. UFBP1 was identified alongside UFL1 in UFM1 co‐immunoprecipitation and mass‐spectrometry analyses [24]. Work from our lab employing a yeast‐2 hybrid screening identified UFBP1 as a direct interacting partner of UFL1 [4]. Originally described as a substrate, UFBP1 is now known to play an essential structural role in stabilizing the UFL1‐UFBP1 complex and also in substrate UFMylation [4, 24]. Whereas UFL1 expressed alone is unstable and inactive, co‐expression with UFBP1 yields a stable heterodimeric complex that is able to catalyse substrate UFMylation [4]. Further removal of NTD (N‐Terminal Domain) region of UFBP1 impacts UFMylation of substrates such as MRE11, Histone H4 and RPL26.

**Fig. 5:**
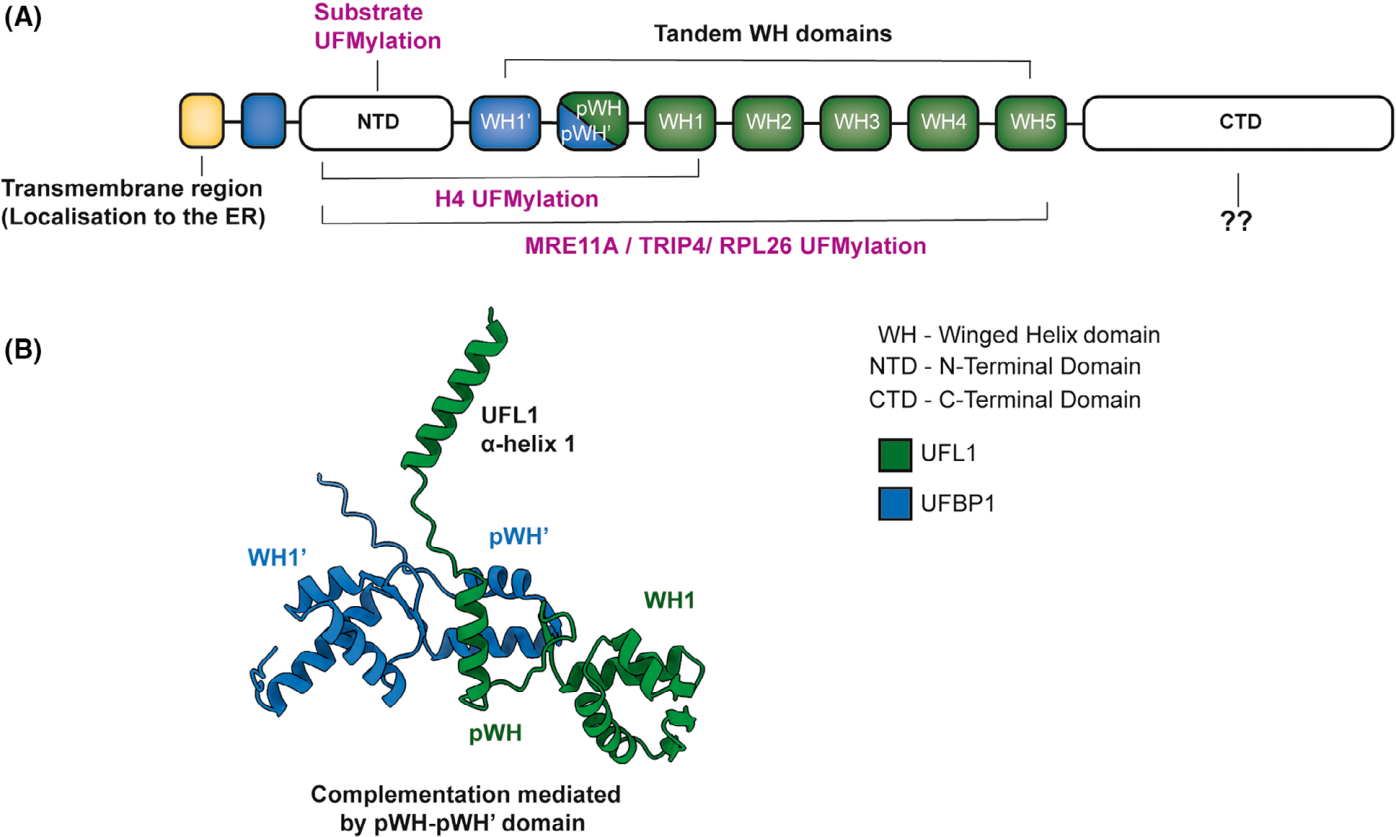
Features of the unusual E3 ligase complex. (A) Schematic showing key domains of the UFL1‐UFBP1 E3 ligase complex. Regions contributing to various functions of the E3 ligase complex are highlighted. NTD, N‐terminal domain; CTD, C‐terminal domain. (B) Alphafold prediction of UFL1‐UFBP1 interaction reveals complementary partial Winged‐Helix (pWH) domains at the interface. The predicted structure of the minimal catalytic domain formed by tandem WH domain and a helix extension of UFL1‐UFBP1 complex is shown in cartoon representation [4] generated using ucsf chimerax [91, 92].

In the absence of structural insights and analogy to ubiquitylation, the essentiality of UFBP1 required a more nuanced analysis. Alphafold structural predictions revealed an explanation for UFL1‐UFBP1 interdependence that was not immediately clear from analysis of the amino acid sequence [4]. Unexpected structural features of UFL1 include five consecutive proteosome component (PCI)‐like Wing‐Helix (WH) domains and a sixth partial WH domain. Interestingly, the partial WH domain is made whole by a complimentary region of UFBP1, which possesses a C‐terminal WH and partial WH domain at its interface with UFL1 (Fig. 5). Structure‐guided mutagenesis and biochemical reconstitution support the essentiality of these complimentary partial WH domains, which are the minimal requirement for the expression and purification of stable UFL1‐UFBP1 complexes *in vitro*. The lack of catalytic cysteines in either UFL1 or UFBP1 indicates that the UFL1‐UFBP1 complex functions as a scaffold‐type E3 ligase [4]. This is analogous to the well‐studied RING‐type class of E3 ligases which bind both charged E2 and the substrate to activate the transfer of ubiquitin [4, 48]. Further structural studies are required to elucidate how this unusual E3 ligase complex recognizes its substrate and how it activates the E2 for transfer of UFM1.

## Substrates of protein UFMylation reveal cellular functions

A limited number of UFMylated proteins have been identified to date that include RPL26 (uL24), RPS20 (uS10), RPL10 (uL16), eIF6, RPN1, UFBP1, UFC1, p53, MRE11, Histone H4, ASC1, and CYB5R3 [3, 13, 16, 18, 24, 49, 50, 51, 52, 53, 54]. This relatively small substrate repertoire reflects the specific nature of the UFM1 pathway compared with ubiquitylation [55]. Recent studies have reported the ribosomal subunit RPL26 to be one of the main cellular targets of UFMylation [16]. This ribosomal subunit is located adjacent to the exit tunnel and close to the Oligosaccharyl Transferase (OST) and SEC61/62/63 complexes that associate with the ribosome during co‐translational folding of nascent peptides [16, 56, 57]. This proximity to the ribosome‐ER interface has led to the suggestion that it may be physiologically relevant to phenotypes and morbidities attributed to the UFM1 pathway, discussed in a later section [16]. RPL26 UFMylation can be induced specifically at the ER upon translational stalling either upon treatment of cells with Anisomycin, an antibiotic that inhibits peptidyl transfer, or by the expression of an ER stalling reporter containing a poly(A) stretch downstream of an ER‐targeting signal sequence [18]. Further, several groups have explored RPL26 UFMylation using siRNA depletion or CRISPR‐Cas9 mediated genetic manipulation of cell line models to reveal both expected and intriguing phenotypes. While loss of UFL1 or its interaction with UFBP1 abolishes RPL26 UFMylation, *UFSP2* ^*−/−*^ cell lines exhibit a striking increase in RPL26 UFMylation on two distinct lysine residues (K132, K134) [16]. This modification is sequential as over‐expression of RPL26^K134R^ in *UFSP2* ^*−/−*^ cells abolishes both mono‐ and di‐UFMylated RPL26, while in cells over‐expressing RPL26^K132R^, mono‐UFMylated RPL26 is still observed [16]. In circumstances where UFMylation is sub‐optimal, such as in UFSP haplo‐insufficient cell lines (*UFSP1* ^*−/+*^/*UFSP2* ^*−/−*^), only K134 modification is observed. These data suggest a preference for K134 modification in the first instance [13]. RPL26 modification is also highly specific as the replacement of endogenous *RPL26* with a mutant allele lacking both residues (RPL26^K132R/K134R^) eliminates RPL26 modification [16].

The localization of the E3 ligase complex to the ER makes it likely that UFMylation mainly functions at the ER. RPL26 UFMylation has been suggested to trigger the lysosome‐mediated depletion of ER‐resident proteins via a process termed ER‐phagy, further indicative of UFMylation in the clearance of aberrant or misfolded proteins in circumstances where the translational process is impeded [12, 49]. This pathway operates independently of Endoplasmic Reticulum‐Associated protein Degradation (ERAD) and likely serves a distinct function that may include the clearance of partial nascent peptides obstructing RQC [16, 49]. Such ideas are consistent with the documented phenotypes related to ER‐stress in cell lines lacking UFM1 pathway components or in phenotypic observations of UFM1‐linked morbidities that are plausibly ER‐stress driven (e.g. cardiomyopathy, defective haematopoiesis and skeletal dysplasia) [47, 58, 59, 60, 61, 62].

In addition to RPL26, recent studies have identified other UFMylated proteins in cells. Interestingly many of them are nuclear‐localized and appear to have roles in regulating DNA damage responses and genome stability with the other reported substrates being modified under specific circumstances. For example, in cancer cell lines (U2OS, HeLa and HCT116), the UFMylation apparatus positively regulates protein levels of the tumour suppressor p53 in a process that may involve antagonism with other PTMs. Of note, these studies required the chemical induction of p53 expression using chemotherapeutic agents (etoposide, doxorubixin) [53]. Another example is Histone H4, which is UFMylated as part of the DNA damage response to ionizing radiation [50, 63]. UFMylation is also reported to regulate telomere length via modification of MRE1 [52], and transcriptional regulation by promoting ASC1‐mediated enhancer trans‐activation (P300/CBP recruitment) [3]. These studies point to important roles for UFMylation in maintaining genome stability. Despite compelling evidence in these studies suggesting nuclear roles for UFMylation, UFBP1, which is necessary for UFL1 stability and catalytic activity, is anchored to the cytosolic face of the ER membrane. It is therefore as yet unclear how nuclear UFMylation is catalysed. It is tempting to speculate that UFL1 may partner with hitherto unidentified hemi‐WH domain‐containing proteins in the nucleus to function as an active E3 ligase. Alternatively, it is suggested that UFBP1 may localize to the nucleus via a putative nuclear localization sequence [62].

## The puzzle of CDK5RAP3 – substrate adaptor or ligase component?

Work by several groups has identified CDK5RAP3 (also referred to as LZAP and C53) as a UFL1‐associated protein [4, 62, 64, 65]. Resolving the function of CDK5RAP3 has proved challenging. When added to *in vitro* reconstitution assays, CDK5RAP3 has an acute inhibitory effect on the formation of UFMylation products and prevents the conjugation of UFM1 to substrates *in vitro* (TRIP4, Histone H4). Further, CDK5RAP3 inhibits aminolysis by UFC1, suggesting that it may function to inhibit UFMylation [4]. However, when purified 60S ribosomes are used as substrates *in vitro*, CDK5RAP3 does not prevent mono‐UFMylation of RPL26 but instead reduces formation of di‐ or tri‐UFMylated species [4]. Do these findings indicate a role for CDK5RAP3 in restricting or otherwise limiting the activity of the enzyme complex to ribosomes? One possibility is that CDK5RAP3 has important functions in directing UFMylation to specific lysine residues on RPL26. At least some observations may support this view. Transient knockdown of CDK5RAP3 in *UFSP2* ^*−/−*^ cells inhibits the formation of di‐UFMylated RPL26 species, while leaving mono‐UFMylated RPL26 intact [16]. A second study reported changes to the UFM1 substrate repertoire based on immunoblotting of whole tissue liver lysates from hepatocyte‐specific *Cdk5rap3* ^*−/−*^ mice leading to suggestions that CDK5RAP3 could function as a substrate adaptor restricting UFMylation to bona fide substrates [15]. However, this study predates much of the recent work on RPL26 and does not determine the identity of affected substrates. Despite being a hepatocyte‐specific knockout, these animals die prematurely (~ 2 weeks after birth), possibly due to growth and developmental abnormalities stemming from defective liver function. Of note, these hepatocytes lacking CDK5RAP3 showed elevated phospho‐Perk, phospho‐eIF2α, and Xbp‐1 cleavage, suggesting ER‐stress to be a likely contributing factor to premature death [15]. Another study reports activation of the unfolded protein response (UPR) and ER‐enlargement in U2OS cells lacking CDK5RAP3 [66]. These ER‐stress phenotypes align with observations of other UFM1 pathway components and suggest an essential role for CDK5RAP3, even if the nature of that role remains enigmatic.

A recent study in plants suggests a further possible role for CDK5RAP3 as an autophagy receptor that is recruited to stalled ribosomes through its association with UFL1. Here, CDK5RAP3 complexes with ATG8 via an unconventional ATG8‐interacting motif (AIM) to initiate autophagy. UFM1 and ATG8 may compete for three conserved AIM‐like sequences in the intrinsically disordered region (IDR) of CDK5RAP3, wherein UFM1 blocks CDK5RAP3 interaction with ATG8 to prevent auto‐phagocytosis [67]. This model bridges results from unbiased CRISPR screening and biochemical analysis of mammalian cell lines where UFMylation components were found to regulate ER‐restricted autophagy (ER‐Phagy) distinct from ERAD [16, 49]. It is therefore suggested that, through a CDK5RAP3‐ATG8 pathway, UFMylation may support clearance of nascent chains that become ‘stuck’ following ribosome stalling [12]. As discussed here, the function of CDK5RAP3 is one of the most intriguing areas requiring further study.

## Insights from murine models

Studies have linked the UFM1 pathway to both innate and adaptive immune responses. These studies take advantage of conditional genetic systems, for tissue‐restricted deletion of UFM1 pathway components. This is necessary as knockout mice for UFM1 pathway components (*Ufm1* ^*−/−*^, *Ufl1* ^*−/−*^, *Uba5* ^*−/−*^, *Ufbp1* ^*−/−*^, *Cdk5rap3* ^*−/−*^) are all embryonic lethal at approximately E11.5. Morbidity is attributed to defective erythrocyte development resulting in severe anaemia [15, 68, 69, 70]. Tissue‐specific knockouts have been more informative and reveal a role for UFMylation in maintaining homeostasis at the ER especially in secretory cells. For instance, B‐cell‐specific knockout mouse models (*Ufbp1* ^*fl/fl*^/*CD19* ^*Cre*^) show that plasma B‐cells, a subset of lymphoid resident antibody‐secreting B‐cells, require UFMylation to support their differentiation into secretory cells. Importantly, *CD19* ^*+*^ B‐lymphocytes lacking *Ufbp1* show signs of ER‐stress and its absence inhibits ER‐membrane expansion required to support high translational loads during immunoglobulin secretion. The ER stress phenotype includes activation of the Unfolded Protein Response (UPR) evidenced by elevated phospho‐PERK, IRE1α and ATF4 protein levels, and increased XBP1 splicing (XBP1s). Interestingly, plasma cell development, but not Immunoglobulin secretion or ER‐expansion, can be rescued by combined deletion of PERK (*Perk* ^*fl/fl*^/*Ufbp1* ^*fl/fl*^/*CD19* ^*Cre*^). The regulation of ER‐homeostasis and plasma cell function (Immunoglobulin secretion) is meanwhile attributed to an alternative branch of the UPR pathway activated downstream of IRE1α [71]. Together these findings highlight the complex role of UFMylation in plasma cell development and function. In another example of loss of UFMylation‐linked ER‐stress, liver‐specific knockout of CDK5RAP3 impeded liver development (hypotrophy) and survival. Conditional knockout mice (*Cdk5rap3* ^*tm1d/tm1d*^ */Foxa3* ^*Cre*^) exhibited defective glucose metabolism, were abnormally small, and typically died within the first 3 months of life. These phenotypes are accompanied by hallmarks of ER‐stress [15].

UFMylation also has essential roles in the proper development and function of the central nervous system (CNS). Deletion of UFM1 in the central nervous system (*Ufm1* ^*fl/fl*^/*Nestin* ^*Cre*^) impairs brain development and is lethal within the first day of life [46]. Observations of *uba‐5* knockout in *C. elegans* identifies roles in cholinergic neurotransmission [72]. Our final example relates to the gastrointestinal tract. The specific deletion of *Ufl1* in Intestinal Epithelial Cells (IECs) (*Ufbp1* ^*fl/fl*^/*Villin* ^*Cre*^) causes tissue‐wide perturbations to homeostasis with histological examination suggestive of increased apoptosis leading to reduced numbers of specialized intestinal cell types (Paneth cells, Goblet cells). In the DSS colitis model of colitis, *Ufbp1* ^*fl/fl*^/*Villin* ^*Cre*^ mice show increased pro‐inflammatory cytokine (IL‐6, IL‐1β) production and are sensitive to tissue damage [73]. These tissue‐specific deletions highlight the requirement of UFMylation in different cell types contributing to tissue and organismal homeostasis.

## Relevance to infectious diseases

A recent genome‐wide CRISPR‐Cas9 screen identified an unexpected role for UFMylation in the antiviral host response. The replication of hepatitis A virus was reduced in cell lines lacking UBA5, UFM1 or UFSP2, demonstrating a requirement for UFMylation in viral replication. This effect was dependent on ribosome modification as ectopic over‐expression of UFMylation‐defective RPL26 similarly reduced viral replication. UFMylation of RPL26 was not detectable by immunoblot analysis indicating a transient or low‐level requirement for UFM1 modification of ribosomes [74]. Other studies have shown that ribosome stalling activates antiviral immune responses via interferon production downstream of the cGAS‐STING pathway [75]. Activation of UFMylation, or perhaps the RQC machinery more broadly, may therefore, connect the translational apparatus to pattern recognition receptor defence mechanisms to prevent viral exploitation of the cellular machinery. Interestingly, mRNA and protein levels of *Ufl1* are rapidly down‐regulated in peritoneal macrophages following infection with Herpes Simplex Virus (HSV‐1). Macrophage‐specific *Ufl1* ^*−/−*^ mice (*UFl1* ^*fl/fl*^ *Lyz* ^*cre+/−*^) experienced an increased viral load and reduced levels of pro‐inflammatory cytokines (IL‐6, TNF‐α, IFN‐β) in serum and peripheral immune cells examined post infection [76]. Intriguing UFL1 directly interacts with cGAS‐STING to mediate these effects [76]. Similarly, roles for UFMylation have been identified in RIGI signalling, or in IFN‐γ‐mediated macrophage priming, which may also contribute to impaired immune responses observed in mouse models [77, 78]. Whether UFM1 involvement in viral pathogenesis is due to effects on viral replication or a direct role in innate immune signal transduction will require further study. Given that UFMylation regulates global proteostasis, a pertinent question is whether these observations reflect specific functional involvements of UFM1 substrates or an overall immunological defect resulting from reduced capacity of the translational apparatus. Nevertheless, these studies suggest that the UFM1 system may be an attractive drug target to block viral infection or to dampen immune responses in cases of autoimmunity and inflammation.

## Relevance to human physiology

Clinical studies documenting morbidities with genetic links to UFM1 pathway components point to essential roles in development and tissue homeostasis. Mutations in most UFM1 pathway components have been linked to a type of bone pathology termed Sohat‐type spondyloepimetaphyseal dysplasia (SEMD). A clinical case study of an affected Italian family reported a missense mutation in the UFSP2 catalytic triad (UFSP2^D418A^). Patients are reported to exhibit infantile‐onset systemic skeletal dysplasia, delayed bone development, demineralization and atrophy, restricted mobility and joint destruction [58]. The SEMD phenotype has also been observed in individuals with mutations in the catalytic histidine (H420R) and the tyrosine residue forming the oxyanion hole (Y282H) [59, 79]. The Y282H mutation results in a notably milder phenotype that reflects the non‐critical function of this residue [59]. Analyses of equivalent residues in UFSP1 (Y41H) suggest that mutation of the tyrosine residue required for oxyanion hole formation simply reduces the rate of proteolysis [39]. These disease mutations suggest that not only the magnitude, but also the timing of UFMylation, is important for function.

Intriguingly, other loss of function mutations that occur outside of the catalytic domain of UFSP2 cause completely unrelated pathotypes. A V115E substitution in the UFSP2 N‐terminal region results in severe neurodevelopment pathology including epilepsy, atrophy of the brain, immobility and severe cognitive impairment [80]. This mutation leads to reduced protein levels of UFSP2 and occurs in one of three parallel β‐sheets that is at the UFSP2 interface with ODR4 [13]. Given that ODR4 is important for stabilizing UFSP2 protein levels in HEK293 cells (and *vice versa*) [13], interference with this interaction may account for reduced UFSP2 levels in affected individuals [80]. Together, these studies indicate that mutations affecting UFSP2 catalytic activity have completely different biological consequences compared with mutations affecting protein levels of UFSP2 [58, 59]. This may suggest additional structural or scaffolding contributions of UFSP2/ODR4 that is critical for the function of the UFMylation apparatus. Alternatively, if the UFSP2^V115E^ mutation affects protein levels of ODR4, then neurodevelopmental defects may reflect functions of ODR4 that are independent of UFMylation. Indeed, an early report describes an adapter function of ODR4 in ensuring the localization of odorant receptors to the cilia of olfactory neurons [81].

Somewhat similar though not identical observations have been made of patients with mutations in other UFM1 pathway components including UFM1, UBA5, and UFC1, which cause encephalopathy in patient groups with overlapping clinical manifestations that include microcephaly (abnormally small cranial development), seizures, progressive emaciation, weakening and rigidity of muscle tissues [47]. The mutations underlying these pathotypes are described as hypomorphic with the knowledge that complete loss of function would likely result in early developmental lethality. How do we explain these pathotypes at the cellular level? A study of patients with mutations in UFBP1 provides some insights where the authors note a striking similarity to pathotypes resulting from defective type‐2 collagen (COL2A1) synthesis. Studies using morpholino knockdown in zebrafish together with post‐mortem analysis of *Ufbp1* ^*−/−*^ mice, indicate that Ufbp1 is essential for early embryonic cartilage development. Reduced *Col2a1*/*col2a1* gene expression is a feature of both experimental models and may be responsible for overlapping clinical manifestations of patients with SEMD and those with diseases classically attributed to type‐2 collagen defects [82]. These findings echo observations made in omics profiling of *UFM1* ^*−/−*^ cell lines where abnormal gene expression of various collagens and other ECM‐linked genes featured prominently [16]. These data are in agreement with global proteomics analysis of *UFSP1* ^*−/−*^, *UFSP2* ^*−/−*^, and *UFSP1/2* ^*−/−*^ cell lines that support a hitherto unappreciated role for protein UFMylation in tissue homeostasis thereby explaining UFM1 involvement in SEMD [13]. Further studies are required to understand the mechanistic basis for UFM1 contributions to tissue homeostasis and bone growth (Table 1).

Based on the impact of mutations in UFMylation components in several diseases and data from murine studies and cellular models, we suggest possible mechanisms by which UFMylation may maintain cellular homeostasis: (a) UFM1 is required either for quality control of secretory proteins or to support a collagen‐specialized secretory pathway; (b) UFM1 modification occurs at a subset of ribosomes responsible for the synthesis of collagen and related proteins (functional specialization). Similarly, UFMylation may have particular relevance to ribosomes at specific stages of human development or in a limited set of tissues [83]; (c) UFMylation regulates ribosome biogenesis and/or recycling of ribosomes; and (d) UFMylation of other substrates, i.e. non‐ribosomal and non‐ER associated proteins to regulate other pathways.

## Concluding remarks

UFM1 has evolved from an enigmatic posttranslational modification to one of the most intensely studied ubiquitin‐like proteins. This is long overdue as the UFM1 pathway components are essential for life. Recent work identifies roles for UFMylation in ER‐ribosome homeostasis where it may support the RQC machinery by facilitating the lysosomal clearance of misfolded or partial nascent peptides [12, 13, 16, 29]. Despite these advances, several questions remain about how UFMylation maintains ER homeostasis. Some of these questions include the signals triggering UFMylation, the identification of factors that recognize UFMylated ribosomes, how UFMylation and deUFMylation are orchestrated and how nuclear substrates are UFMylated. We believe that in addition to studies aimed at dissecting the cell biology of this pathway, mechanistic studies of how ribosomes are UFMylated will reveal the functional importance of UFMylation. Meanwhile, complementary translational studies on the organism‐level contributions of UFM1 pathway components will highlight clinical opportunities arising from the studies we have described.

## Conflict of interest

The authors declare no conflict of interest.

## Author contributions

All authors contributed to the preparation of this manuscript. DM wrote the first draft of the manuscript while JJP prepared the figures.
